# Initiating an undiagnosed diseases program in the Western Australian public health system

**DOI:** 10.1186/s13023-017-0619-z

**Published:** 2017-05-03

**Authors:** Gareth Baynam, Stephanie Broley, Alicia Bauskis, Nicholas Pachter, Fiona McKenzie, Sharron Townshend, Jennie Slee, Cathy Kiraly-Borri, Anand Vasudevan, Anne Hawkins, Lyn Schofield, Petra Helmholz, Richard Palmer, Stefanie Kung, Caroline E. Walker, Caron Molster, Barry Lewis, Kym Mina, John Beilby, Gargi Pathak, Cathryn Poulton, Tudor Groza, Andreas Zankl, Tony Roscioli, Marcel E. Dinger, John S. Mattick, William Gahl, Stephen Groft, Cynthia Tifft, Domenica Taruscio, Paul Lasko, Kenjiro Kosaki, Helene Wilhelm, Bela Melegh, Jonathan Carapetis, Sayanta Jana, Gervase Chaney, Allison Johns, Peter Wynn Owen, Frank Daly, Tarun Weeramanthri, Hugh Dawkins, Jack Goldblatt

**Affiliations:** 1 Genetic Services of Western Australia, Department of Health, Government of Western Australia, Perth, WA Australia; 2 School of Paediatrics and Child Health, University of Western Australia, Perth, WA Australia; 3 Institute for Immunology and Infectious Diseases, Murdoch University, Murdoch, WA Australia; 4 Office of Population Health Genomics, Public Health Division, Department of Health, Government of Western Australia, Perth, WA Australia; 5 Telethon Kids Institute, University of Western Australia, Perth, WA Australia; 6 Diagnostic Genomics, PathWest, Department of Health, Government of Western Australia, Perth, WA Australia; 7 Centre for Population Health Research, Curtin Health Innovation Research Institute, Curtin University of Technology, Perth, WA Australia; 8 School of Pathology and Laboratory Medicine, University of Western Australia, Perth, WA Australia; 9 Centre for Comparative Genomics, Murdoch University, Perth, WA Australia; 10 Public Health Division, Department of Health, Government of Western Australia, Perth, WA Australia; 11 Western Australian Register of Developmental Anomalies, Perth, WA Australia; 12 King Edward Memorial Hospital, Perth, WA Australia; 13 School of Spatial Sciences, Curtin University, Perth, WA Australia; 14 Cooperative Research Centre for Spatial Information, Perth, WA Australia; 15 Perth Children’s Hospital, Perth, WA Australia; 16 Kinghorn Centre for Clinical Genomics, Garvan Institute for Medical Research, Darlinghurst, NSW Australia; 17 St. Vincent’s Clinical School, Faculty of Medicine, University of New South Wales, Darlinghurst, NSW Australia; 18 The Children’s Hospital at Westmead, Clinical Genetics Service, Westmead, NSW Australia; 19 National Human Genome Research Institute, National Institutes of Health, Bethesda, Rockville, MD USA; 20 Undiagnosed Diseases Program, Common Fund, Office of the Director, National Institutes of Health, Bethesda, Rockville, MD USA; 21 National Centre for Advancing Translational Sciences, National Institutes of Health, Bethesda, Maryland, USA; 22 Instituto Superiore di Sanità, National Center for Rare Diseases, Rome, Italy; 23 Canadian Institutes of Health Research, Institute of Genetics, Montreal, Canada; 24 Keio University School of Medicine, Tokyo, Japan; 25 Wilhelm Foundation, Brottby, Sweden; 26 Department of Medical Genetics, University of Pécs, Pécs, Hungary

**Keywords:** Diagnosis, Genomics, Phenomics, Undiagnosed, Diagnostic odyssey, Clinical best practice, Policy, Precision public health

## Abstract

**Background:**

New approaches are required to address the needs of complex undiagnosed diseases patients. These approaches include clinical genomic diagnostic pipelines, utilizing intra- and multi-disciplinary platforms, as well as specialty-specific genomic clinics. Both are advancing diagnostic rates. However, complementary cross-disciplinary approaches are also critical to address those patients with multisystem disorders who traverse the bounds of multiple specialties and remain undiagnosed despite existing intra-specialty and genomic-focused approaches. The diagnostic possibilities of undiagnosed diseases include genetic and non-genetic conditions. The focus on genetic diseases addresses some of these disorders, however a cross-disciplinary approach is needed that also simultaneously addresses other disorder types. Herein, we describe the initiation and summary outcomes of a public health system approach for complex undiagnosed patients - the Undiagnosed Diseases Program-Western Australia (UDP-WA).

**Results:**

Briefly the UDP-WA is: i) one of a complementary suite of approaches that is being delivered within health service, and with community engagement, to address the needs of those with severe undiagnosed diseases; ii) delivered within a public health system to support equitable access to health care, including for those from remote and regional areas; iii) providing diagnoses and improved patient care; iv) delivering a platform for in-service and real time genomic and phenomic education for clinicians that traverses a diverse range of specialties; v) retaining and recapturing clinical expertise; vi) supporting the education of junior and more senior medical staff; vii) designed to integrate with clinical translational research; and viii) is supporting greater connectedness for patients, families and medical staff.

**Conclusion:**

The UDP-WA has been initiated in the public health system to complement existing clinical genomic approaches; it has been targeted to those with a specific diagnostic need, and initiated by redirecting existing clinical and financial resources. The UDP-WA supports the provision of equitable and sustainable diagnostics and simultaneously supports capacity building in clinical care and translational research, for those with undiagnosed, typically rare, conditions.

**Electronic supplementary material:**

The online version of this article (doi:10.1186/s13023-017-0619-z) contains supplementary material, which is available to authorized users.

## Background

Undiagnosed diseases are often rare, sometimes extremely rare. These conditions may represent expanded phenotypes of more frequent rare disorders; also sometimes apparently single diseases may be the phenotypic expression of multiple disorders or an unusual presentation of a more common disease [1]. They may also be due to an underlying truly new disease. In each of these instances the primary aetiology can be genetic or not (e.g., epigenetic or environmental). There are 5,000–8,000 known rare diseases and, because of their individual rarity, achieving a diagnosis can be particularly challenging. In a European study, 25% of individuals waited 5–30 years for a diagnosis and in 40% of instances the initial diagnosis was wrong [2]. Similar findings were demonstrated in Australia; approximately 30% of patients waited for more than 5 years to receive a diagnosis, a similar number saw more than 6 doctors before receiving a diagnosis, and half had at least one incorrect diagnosis [3]. Reflecting a priority for the global rare diseases community, local and international efforts [4–6] have been developed to address this diagnostic odyssey, since an accurate diagnosis is the bedrock of best practice medical care.

Most rare diseases have genetic origins. Hence, technological advances such as chromosomal microarray, followed in more recent years by the clinical application of massively parallel sequencing (primarily exomic sequencing), have resulted in a markedly increased diagnostic yield in rare diseases [7]. International experience with the clinical implementation of exome sequencing has typically shown a diagnostic yield of 25–30%, whereas prior to the use of this technology the yield in some cases was less than 10% [5]. The implementation of additional technological advances such as whole genome sequencing and phenotype-enabled diagnostic analyses [8] will further increase the diagnostic yield, but even so it is likely that at least 50% of patients for whom a diagnosis is sought using these technologies will remain undiagnosed. This means that complementary approaches to “solve the unsolved” are required.

The United States (US) National Institutes of Health (NIH) Undiagnosed Diseases Program (UDP) commenced in 2008 to address the unmet needs of those living with rare or multisystem diseases who remained undiagnosed after exhaustive efforts. For most pediatric patients in the program, the diagnostic odyssey averaged more than 5 years. The UDP had three major objectives: i) to provide an accurate diagnosis ii) to facilitate new disease discovery, and iii) to improve understanding of biological pathways that would lead to a greater understanding of human physiology in rare and more common disorders [1]. The volume of undiagnosed patients applying to participate in the program was overwhelming and underscored the magnitude of the unmet need.

Technological advances in genomics, and other –omics, are key enablers of the UDP. However the most important factor is the involvement of multiple rare diseases experts, largely through consultations during UDP admissions [1]. In the UDP, approximately half of the diagnoses were made directly from disease agnostic, but phenotype informed, genomic testing through massively parallel sequencing. The remaining diagnoses were made by focused biochemical, radiologic, and molecular studies suggested by rare disease experts given the opportunity to investigate the problem as a collaborative team. The UDP has a core power of focusing assessments and expertise in one place at one time and centered on one patient at a time; it harnesses the richness of face-to-face discussion and real-time and in-person clinical assessments.

The UDP paradigm and nexus expanded to an extramural network of nodes at leading clinical academic centers in the US, i.e., the Undiagnosed Diseases Network (UDN) [9]. Subsequently, the Undiagnosed Diseases Network International (UDNI), an expanding international network operating under the same core principles, was formed [4]. The set up of UDNI was preceded by a number of formative meetings in Europe (Italy, Budapest and Vienna) and Japan (Tokyo) and a workshop in Perth, Western Australia [4]. The Perth workshop aimed to identify the key success factors and challenges to implementing a UDP in Australia as a means to find diagnostic solutions for intensive users of clinical services.

Following the Perth workshop, the UDP-WA was conceived and implemented as a program within the public health system in March 2016, with links to research partners but not driven from academia. In Western Australia, the public health system is comprised of a Department of Health and four Area Health Services. The Department of Health acts as the system manager and commissions, monitors and evaluates the performance of services provided by the Area Health Services. The public health system can be accessed and used by all residents in Western Australia.

The vision was that the UDP-WA would also support other Australian states and New Zealand to establish UDPs as part of the UDNI. Herein we describe the implementation and initial learnings of the UDP-WA.

## Methods

The first UDNI meeting in Italy sought to share the benefits of the UDP and the UDN and enhance them through partnering internationally. The workshop was sponsored by the US NIH Common Fund and a patient organization, the Wilhelm Foundation, who along with the NIH recognised the importance of international collaborative efforts in tackling undiagnosed diseases. It was primarily attended by European nationals, and included several Australian delegates.

Less than 1 year later, in August 2015, Western Australia hosted the first Australasian UDP workshop. This was attended by a largely Australian audience, with a strong local (Western Australian) presence. All Australian jurisdictions (with the exception of the Northern Territory and Tasmania) were represented at the workshop. Importantly, a number of Asian government and research agencies also attended (primarily from Singapore and Malaysia). In all, close to 90 researchers, clinicians, patient groups and representatives of public and private organizations attended.

Workshop attendees heard from founders of the NIH program about running a UDP. Through facilitated discussions, considerable time during the workshop was dedicated to exploring what difference a UDP program in Australia or regional countries would make to patients, clinicians and health systems. The challenges and benefits of implementing a UDP were also explored. Ultimately, the recognised benefits motivated attendees and ensured support for the commencement of the UDP in WA. Recognition of these benefits also fostered a strong interest in the UDP model elsewhere in Australia, and helped strengthen a national network of clinicians and researchers working in the area of rare diseases.

A range of challenges that implementing a UDP could present were discussed in this Workshop. Notable among the issues raised was the difficulty posed by operationalizing a UDP within a public health system as a clinical entity using existing resources. This issue is compounded as the health system model has a significant focus on individual specialties and health services which can complicate their interaction. This in turn raised the challenge of capturing the broad range of clinical knowledge required to enable the program to work; an integral function of a UDP. Other key concerns were questions on how the program could be provided equitably such as reaching patients in regional WA, and also how it could be sustainable. Many of the innovative ways in which these challenges have been met are described within this paper.

The workshop also comprehensively explored the positives a UDP could bring. An immense volume of potential positive outcomes attendees thought could accrue from delivering a UDP were raised. Three broad areas of benefit were distilled from the conversations being’improved health system outcomes’, ‘improved patient outcomes and ‘enhanced knowledge sharing information’. Within each of these broad areas a range of potential benefits was identified. For example, within: (i) improved health system outcomes: increased efficiency through reduced duplication of testing, reduced waitlists, better referral pathways, and better treatment; better allocation and coordination of resources that has greater alignment with patient need. (ii) improved patient outcomes: for those diagnosed through the program, diagnosis could lead to earlier treatment and appropriate management and reduced stress associated with not knowing condition. For those remaining without a diagnosis a sense of closure for the patient and their family. (iii) enhanced knowledge sharing information: for diagnosed families the ability to inform other family members of the condition and the genetic (where relevant) implications for them; for UDP clinicians, greater connection, collaboration and coordination with other clinicians.

Building on the momentum and discussion outcomes of the workshop, local clinical geneticists and policy-makers employed within the Western Australian public health system championed the idea of a UDP in Western Australia. They initiated intensive engagement and relationship building with clinicians at Western Australia’s children’s hospital to promote the development of a UDP. Heads of clinical specialty departments were initially approached for support, which they gave in principle. Higher executive-level support from the hospital and Department of Health was then obtained. Following this, clinicians within the clinical specialty departments were approached to participate in the program as part of a cross-disciplinary team. Support from these clinicians (some of whom are also Department Heads) represents an enormous generosity in the form of donated time, since delivery of the UDP-WA has occurred within existing budgets. Some departments nominated one clinician and others created a roster to share the experience. Still others rearranged their private clinics to participate in this program delivered within the public health system.

## Results

In November 2015, the Department of Health confirmed that the UDP-WA would commence in 2016 [10].

The UDP-WA is coordinated by Genetic Services WA, which delivers statewide genetics services to all residents of Western Australia, and is a department within the North Metropolitan Area Health Service. The UDP-WA has an initial focus on children. Eligibility criteria include those who are: (i) generally at least 6 months old; (ii) have chronic, complex and typically multisystem diseases; (iii) are well known to the public health system, specifically the children’s hospital and the multi-disciplinary UDP-WA team of clinicians; (iv) have typically had multiple specialist assessments and hospital admissions; and (v) have clinical factors supporting the possibility of obtaining a diagnosis with current approaches, yet remain undiagnosed (e.g., multiple affected family members, consanguinity, highly unique phenotypic combinations, facial dysmorphism, growth disturbances e.g., short or tall stature, and microcephaly).

Patients who meet these criteria are reflective of those with the highest need within the undiagnosed community. The UDP-WA is being delivered using existing resources meaning the pace of implementation needs to be manageable and cognisant of other work commitments of the team delivering the program. At this stage, the UDP-WA admits one patient per month and does not accept external referrals.

### UDP-WA Team

This team consists of core roles: Program Director, Program Coordinator, Genetic Counsellor and Expert Panel. The Program Director oversees the program and is responsible for its clinical management. The Coordinator is the point of contact with families and helps to arrange appointments for tests and investigations during the program’s Clinical Stage. Genetic Counselling is provided to families throughout the program. The Expert Panel is a multi-disciplinary team made up of specialists from a range of medical fields. The role of the Expert Panel is to review existing medical history of program patients and make recommendations for further clinical assessment. The Expert Panel is purposely composed of a diverse group of physicians, for instance covering clinical genetics, neurology, imaging, endocrinology, gastroenterology, cardiology, haematology, ophthalmology, respiratory medicine, metabolic medicine and others. All panel members receive patient summaries and can comment on these. Irrespective of the clinical domains identified in each patient and given the typically multisystem presentations, any and all of the specialists are encouraged to contribute and to attend panel meetings to provide a diverse range of perspectives that encourages broad and lateral thinking, and ultimately for triangulation on diagnostic possibilities.

### Clinical flow

**Fig. 1:**
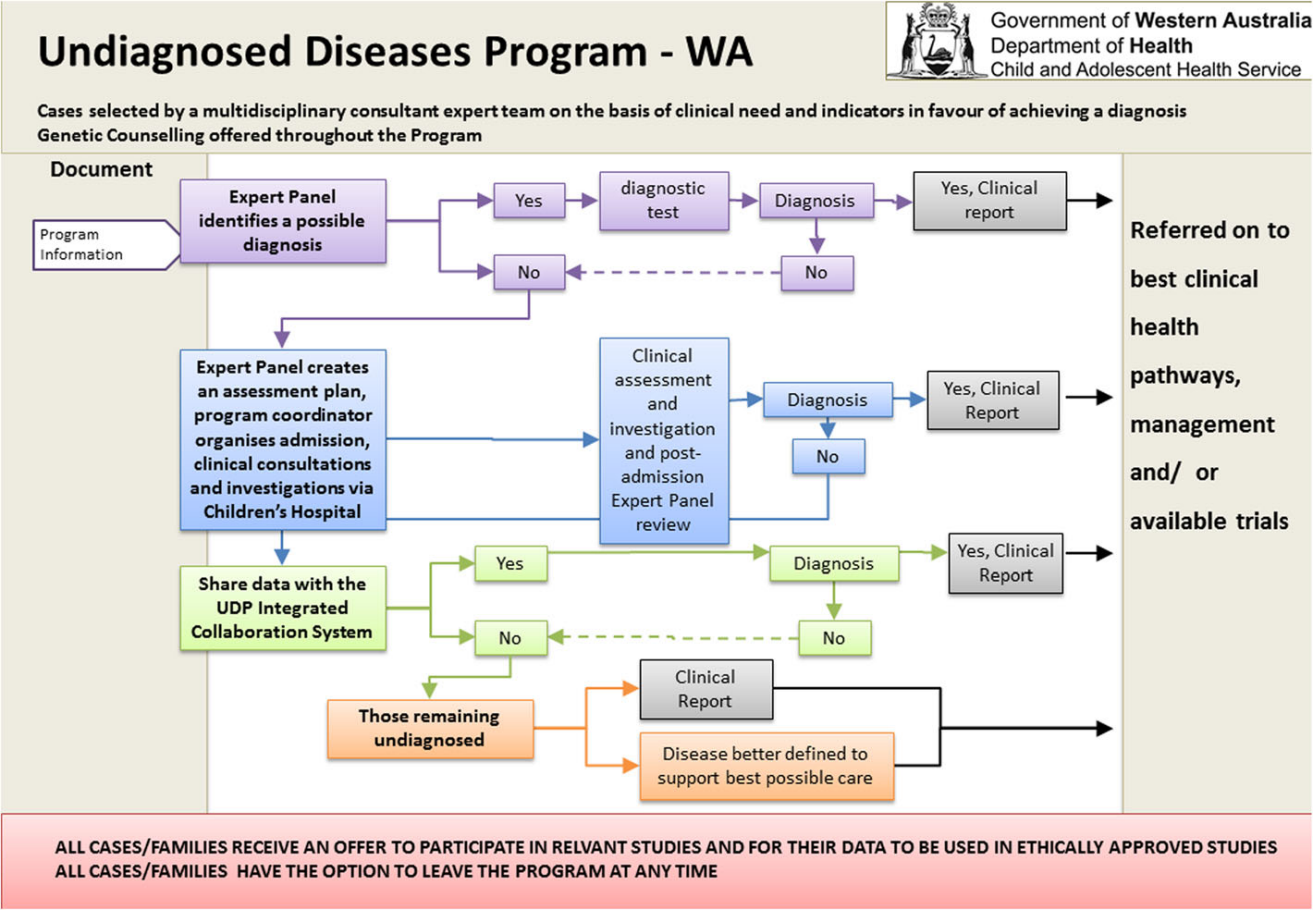
Program Schematic. This schematic represents the Clinical flow of the Undiagnosed Diseases Program WA

To provide structure and a sense of coherence and direction for the new program, a schematic of a clinical pathway was developed (see Fig. 1.)

### Submission template and distribution of summaries

The NIH has shared copies of protocols used in the implementation of their UDP program and these have been adapted for local conditions.

The patient submission template is a key summary document guiding the Expert Panel review of each patient’s case (see Additional file 1). As highlighted by Tifft and Adams [1], the cumulative medical record for UDP patients is often several hundred pages in length and there is substantial work involved in getting this prepared for review; this has proved no different in the WA case. The summary is developed by the clinician responsible for identifying the child as a potential UDP participant (called the referring clinician). The summary includes text and also photographs of the child and relevant family members. Triaging occurs via the Program Director, with the opportunity for consultation for consensus with the Expert Panel members.

The Program Director also reviews all available medical records, including all hard copy records and electronically available investigations and makes modifications to the submission template accordingly. The submission template is distributed to the Expert Panel 1 week before their review meeting to allow initial thoughts to be generated and to allow a forum for broad contribution, including from those who cannot attend at the specific Expert Panel meeting relevant to that child.

Presentation of accurate and up to date information in the submission template, such as the timing of tests and their results, is critical for a robust informed discussion of each case and to guide investigations. The submission template forms the basis for an evolving documentation of diagnostic suggestions, planned investigations and their results, diagnosis (when achieved), and suggestions for further assessments or changes in management.

### Patient flow, assessments and investigations

Following the development of the submission template and selection by the Program Director of patients to participate in the program, the UDP-WA is being delivered to patients in seven key stages.

#### Stage 1

Parents/caregivers of children are invited to take part in the program and following their acceptance, they have a conversation with the Program Coordinator (a genetic counsellor) to learn about the program.

#### Stage 2

The Expert Panel meets to review the case of the program participant. During this meeting they generate a differential diagnosis and map out a series of clinical assessments and investigations they believe will optimise the chance of achieving a diagnosis.

#### Stage 3

Patients attend a day facility at the children’s hospital for up to 5 days where they undertake the range of clinical assessments and investigations set out by the Expert Panel. On occasion, a diagnosis has been made through stages 1 and 2, thus obviating the need for this stage. Stages 2 and 3 provide the platform for capturing the power of a coordinated effort of a group of multiple experts centered around one patient at time, in the same place, and at the same time. This provides the means to realise benefits that may not be achieved through sequential individual assessments. It also captures nuances that are not necessarily communicated through written notes.

#### Stage 4

With the consent of the patient/family, the UDP-WA Program Director shares de-identified phenotypic and genomic data about the child with national and international partners to maximise the opportunity for finding a diagnosis. To enable this, data can be deposited into the Undiagnosed Diseases Program Integrated Collaboration System which is a collaborative workspace for the UDNI.

#### Stage 5

The UDP-WA team determines whether a definitive diagnosis can be found at this stage.

#### Stage 6

Parents/caregivers of children in the program attend a meeting with the Program Director to discuss the findings.

#### Stage 7

A report is written for patients that outlines the definitive diagnosis that was achieved (if any), important new phenotypic findings and test results and suggestions for further assessments and changes in care, which include facilitating connections with relevant support groups or patient communities.

### Genomic testing

Genomic analyses are performed in Stage 3 of the progam, with parallel and complementary approaches. PathWest Diagnostic Genomics undertakes phenotype-driven in-silico filtered whole exome sequencing, as described previously [5]. The Garvan Institute through its clinical testing arm, Genome One, undertakes whole genome analysis using Illumina TruSeq DNA library preparation™ on Hamilton automated liquid handling systems, the Illumina HiSeq X DNA™ sequencing system and a mixture of open source and in house data analysis pipeline and variant filtering [11]. Typically this involves trio analysis of an affected child and unaffected parents, but this can be modified depending on the pedigree e.g., if there are multiple affected family members.

### Data sharing

Sharing patient data as part of the UDP-WA is critical aspect of the program that increases the opportunity of finding diagnoses for patients, and that allows their data to potentially help diagnose patients in other jurisdictions. Data sharing with the UDNI is an evolving process which involves, in the first instance, a commitment to sharing a minimum set of data including human phenotype ontology terms and variants in candidate genes. The UDP-WA has access to the Undiagnosed Diseases Program Integrated Collaboration System (UDPICS) which is a collaborative workspace for undiagnosed diseases patients. Data can also be shared via secure online, internationally utilized repositories such as Phenome Central, which also enables matchmaking. Matchmaking refers to the process whereby a patient’s phenotype and/or genotype can be used to find patients with similar phenotypes and/or genotypes i.e., matched, to enable a diagnosis to be found. Presently this is being supported in Western Australia through the use of the clinical service use of the knowledge management platform, Patient Archive (PA). PA is interoperable with matchmaking pathways, for example it is able to provide data to Phenome Central. In Japan, sharing data involves the use of the Patient Archive architecture, under the name Initiative on Rare and Undiagnosed Diseases (IRUD) exchange. An interoperable ecosystem of approaches for patients with Undiagnosed Diseases continues to evolve.

### Illustrative cases

#### Case 1

The first child admitted to the UDP-WA program was a 7 year old girl with a congenital multisystem disorder. She had nearly 50 hospital inpatient admissions, 13 general anaesthetics and more than 200 hospital outpatient visits for consultations across multiple specialist clinics. She had been presented to national and international experts, the latter including a virtual international expert dysmorphology network. Discussions at the Expert Panel meeting included multiple specialists suggesting the possibility of various RNA processing disorders. A literature review by panel members that evening narrowed the search to Trichohepatoenteric syndrome, a condition with a prevalence of approximately 1 in 1 million. Subsequently compound heterozygous *SKIV2L* mutations were identified by re-analysis of exome sequencing data. Because of the diagnosis the family has been referred into the appropriate management pathways, with the potential to reduce unnecessary and expensive services, and provides the family and medical system with increased certainty. It also allowed the family to connect with other families for support and to reduce isolation.

#### Case 2

The second child admitted to the UDP-WA program was an 11 year old girl with a congenital multisystem disorder associated with short stature, an aesthetic habitus and progeroid facies. Amongst the possibilities discussed by the Expert Panel was an exon 64 *Fibrillin-1* mutation*.* This had been considered previously and assessed by monogenic sequencing and by *in-silico* filtered targeted exome. Following the panel meeting, exome data were reviewed including with a focus on new candidate diagnoses raised during the meeting. No mutation was identified. Given the suggestion by multiple panel members of a marfanoid condition, and despite a normal echocardiogram 2 years previously, a repeat echocardiogram was organized during her admission. This demonstrated aortic root and pulmonary artery dilatation. Also, a repeat skeletal survey showed the new finding of acro-osteolysis. No mutation in known acro-osteloysis-associated genes was detected in the exomic data. Trio whole genome analysis is pending. Whilst no definitive diagnosis has yet been confirmed, the UDP-WA assessment uncovered a highly significant manageable phenotype (aortic dilatation).

## Discussion

We have described the conceptualization and implementation of the Undiagnosed Diseases Program-Western Australia (UDP-WA). This clinical service program, offered within a public health system, was developed to address the unmet needs of those on a protracted diagnostic odyssey. It is a cross-disciplinary initiative targeted to those with particularly complex multisystem disorders, with (when required) an inpatient component. It is a supplement to and synergistic with, an existing outpatient clinical diagnostic pipeline provided through one specialty [5].

The UDP-WA has been implemented following a process of national, regional and international multi-stakeholder engagement. It is being delivered within a public health system to: build on existing state-wide initiatives within that system that facilitate earlier and more accurate diagnosis; allow engagement of world standard clinical specialists across multiple disciplines; integrate with existing clinical flow across the breadth of specialties involved; and allow for geographically and financially equitable access to the program by undiagnosed patients.

Even at this early stage, the UDP-WA is delivering a number of key outcomes, namely:

1. Diagnoses including those confirmed on genomic or phenotypic grounds.
2. There has been refinement of management for patients with and without a diagnosis, such as that resulting from the detection of aortic dilatation in illustrative case 2.
3. Provides a platform for capacity building in diagnostic approaches, including phenomics and genomics that concurrently informs and learns from the multiple specialties that are engaged. This supports simultaneous and efficient cross-disciplinary mainstreaming of these approaches.
4. Supports clinical training through the attendance of junior medical staff. The junior staff have the opportunity to learn from the individual and cumulative wisdom of the senior specialists as they discuss the investigation and management of extremely high acuity cases.
5. Promotes workforce retention and engagement, with senior and/or retired clinicians providing the benefit of their unparalleled wealth of experience.
6. The Expert Panel review meetings allow for discussions centered around one patient at one time, in one place. This captures the nuances of clinical assessments that are not necessarily evident in written communication.
7. Provides a further pipeline to local, national and international translational research, and related training in clinical research.
8. It has fostered the engagement of medical students in rare and undiagnosed diseases, including connection to Students4Rare Diseases [12] and is contributing to the rare diseases knowledge base through crowdsourced curation of disease data including, but not limited to, Gene Wiki [13].

A recurrent and predominating theme expressed by the families participating in the UDP is the psychological benefit of connectedness. This is particularly the case for those with a definitive diagnosis who are connected to the shared experience and support of other families living with the same condition. However, it can also be the case for those who remain undiagnosed given the expanded network of tailored support for Undiagnosed patients and families through advocacy organisations such as: Syndromes Without a Name, United Kingdom [14] and Australia [15]; the “Undiagnosed Diseases Community” of Rare Connect [16], EURORDIS; and the Rare and Undiagnosed Network, National Organisation for Rare Disorders [17]. This feeling of connectedness also extends to the staff participating in the program who are united in purpose through new hope for the patients and families they serve.

The process of conceptualizing and implementing this program has drawn media and philanthropic attention and stimulated new precision medicine research initiatives. It has also supported the uptake of the UDP across other Australian sites. An Australia-New Zealand Undiagnosed Diseases Program Executive Committee has been formed and several jurisdictions are in various stages of planning or implementing UDP programs. Staff from Westmead Children’s Hospital and the Garvan Institute of Medical Research recently hosted a workshop to discuss implementing a UDP in the state of New South Wales [18] and the Victorian Clinical Genetics Service has initiated their program.

Planned further work includes the expansion of the UDP-WA to include adult patients referred to the program by members of the Expert Panel. When this becomes successful, the program will then progress towards accepting external referrals from other clinicians. A community advisory panel will also be established. Additionally, a longitudinal study following parents/caregivers of children going through the program is currently underway as are health economic evaluations relating to the UDP-WA. Plans for a staff impact study, that will include a critical examination of some of the emerging benefits mentioned in this paper, are also being explored.

## Conclusion

Eight years after the commencement of the NIH UDP in Bethesda Maryland, a UDP had been established in Western Australia, some 18,000 km away. In this paper we describe the process of implementing the UDP-WA in a public health system in the hope that this will assist other jurisdictions in considering and implementing similar approaches. The UDP-WA is a complementary approach to an existing statewide genomic diagnostic pipeline. We provide some initial results including some foreseen outcomes such as definitive diagnosis and refined management. Also, we describe some less readily anticipated important outcomes including increased staff cohesion, dissemination of genomic and phenomic knowledge, supporting the training of junior medical staff, retaining/re-attracting work force capacity and generating enthusiasm and engagement of medical students and researchers. The multi-stakeholder engagement, including, but not limited to, the support and enthusiasm of clinicians and health system managers involved in delivery of this program has been critical.

## Additional file

Additional file 1: Undiagnosed Diseases Program WA Patient Summary Template. (DOCX 14 kb)

## Abbreviations

GSWA: Genetic Services of Western Australia
RUDDS: Rare and Undiagnosed Diseases Diagnostic Service
UPD: Undiagnosed Diseases Program
WES: Whole Exome Sequencing
