# Supplementary material for: Initiating an undiagnosed diseases program in the Western Australian public health system
Source: Orphanet J Rare Dis. 2017 May 3;12:83. doi: 10.1186/s13023-017-0619-z (PMC5415708; doi:10.1186/s13023-017-0619-z)
Supplement: Additional file 1: — Undiagnosed Diseases Program WA Patient Summary Template. (DOCX 14 kb) [file 13023_2017_619_MOESM1_ESM.docx]

**UNDIAGNOSED DISEASES PROGRAM WA**

**PATIENT SUMMARY**

**Name:**

**DOB:**

**URN:**

**Submitting Clinician:**

Photos of patient:

(please insert below or as a separate attachment i.e. powerpoint)

**Summary of key phenotypic features:**

**SUMMARY OF PREVIOUS INVESTIGATIONS/RESULTS**

| **TEST** | **DATE** | **RESULT** |
| --- | --- | --- |
|  |  |  |
|  |  |  |
|  |  |  |
|  |  |  |
|  |  |  |
|  |  |  |
|  |  |  |
|  |  |  |
|  |  |  |

**Comments**
